# The relationship between organisational characteristics and the effects of clinical guidelines on medical performance in hospitals, a meta-analysis

**DOI:** 10.1186/1472-6963-6-53

**Published:** 2006-04-28

**Authors:** Rob Dijkstra, Michel Wensing, Ruth Thomas, Reinier Akkermans, Joze Braspenning, Jeremy Grimshaw, Richard Grol

**Affiliations:** 1 Centre for Quality of Care Research-117, Radboud University, PO Box 9101, 6500 HB Nijmegen, The Netherlands; 2 Health Services Research Unit, University of Aberdeen, Aberdeen, Scotland, UK; 3 Ottawa Health Research Institute, 1053 Carling Avenue, Ottawa, ON, K1Y 4E9, Canada

## Background

Systematic reviews in various health care settings have demonstrated that different implementation interventions have varying effects. [1,2]. Most interventions to implement clinical guidelines focused on changing professional behaviour, but there is increasing awareness that factors related to the social, organisational and economical context can also be important determinants of guideline implementation[3]. For instance, a recent study on the implementation of screening guidelines in ambulatory settings has confirmed the influence of a number of organisational factors, such as mission, capacity and professionalism[4]. Despite increasing attention to organisational determinants of guideline implementation, research evidence on the relevance of specific factors is still limited. Insight into these factors is important as it can improve the effectiveness of implementation interventions by tailoring interventions to local circumstances. For example, different interventions may be more effective at academic hospitals than at community hospitals.

**Figure 1:**
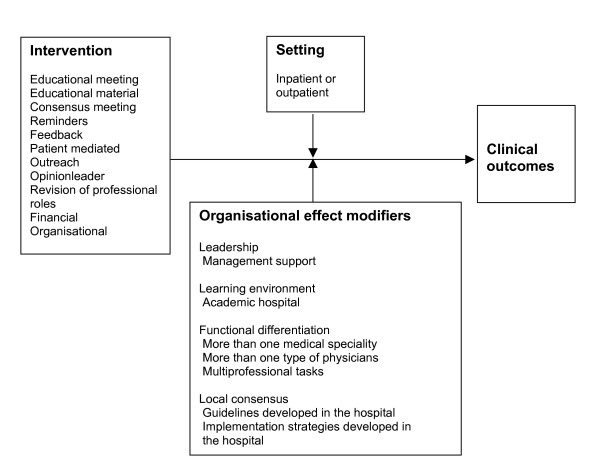
Conceptual framework to assess the relationship between organisational or implementation aspects and clinical outcomes.

Most reviews on guideline implementation were conducted on implementation across settings, or implementation in primary care settings[5]. The literature on guideline implementation in hospital settings has not yet been reviewed separately. Therefore, we reviewed the effect of different intervention strategies to implement clinical guidelines at hospitals, and explored the impact of specific organisational factors on the effectiveness of these interventions. Hospitals are complex organisational systems whose primary aim is to deliver clinical care to individual patients. Management theories on change and innovation were analysed to derive specific factors for this explorative study. We identified the following factors that moght modify the effects of interventions: sufficient management support, appropriate learning environment, functional differentiation and local consensus on the intended changes (figure 1).

Theories on leadership and on quality management have suggested that support for an innovation from hospital management has a positive impact on its adoption [7-9]. The impact of management support may be based on power, incentives or facilitation. Hospital managers may also act as role models by implementing the innovation. Thus we hypothesized that implementation interventions are more effective if the effort is clearly supported by the local leaders.

The learning environment comprises a second set of factors. The underlying mechanism is that the availability of knowledge in the organisation enhances the adoption of innovations. This is consistent with existing theory on organisational learning, which suggests that an organisation's capacity to learn as an organisation is a crucial feature[9]. Teaching hospitals create a specific learning environment for trainers and trainees. Therefore we expected that implementation interventions are more effective in teaching hospitals than in non-teaching hospitals.

Functional differentiation is another factor that is expected to influence the uptake of new information or procedures in practice[10]. A higher level of specialisation and a higher level of technical expertise in the organisation may enhance implementation. The level and diversity of knowledge may be larger in settings with a range of medical disciplines, in which there is involvement of consultants, other physicians and non-physician practitioners. We therefore hypothesized that higher functional differentiation is positively associated with the effectiveness of implementation interventions.

Finally, we expected that promoting ownership through local consensus about clinical guideline recommendations and implementation strategies may also be associated with better uptake[11]. Organisational learning theory suggests that information gathering, shared perceptions of performance gaps and an experimental mind-set are important factors for learning in organisations[9]. Specific group cultures at hospitals appear to be associated with patient outcomes[12]. Theory on complex adaptive systems suggests that innovations should not be specified in detail in order to promote ownership and that 'muddling-through' should steer the guideline implementation process[13], while theory on adult learning adds that implementation should be tailored to each individual's learning needs[14]. We hypothesized that guideline implementation interventions would be most effective when developed within a hospital rather than derived from sources outside a hospital.

This systematic literature review aimed to assess the effectiveness of implementation and quality improvement interventions in hospital settings and to test our hypotheses on the impact of organisational factors.

## Methods

### Inclusion/exclusion

Only studies with a concurrent control group of the following designs were included:

• Randomised controlled trials (RCTs), involving individual randomisation or cluster randomisation on the level of the hospital, ward or professional.

• Controlled clinical trials or controlled before-and-after studies.

Participants: the studies described the performance of medical health care professionals working at the hospitals. Medical centres, health centres or clinics without an inpatient department were excluded. Ambulatory departments and clinics that fell directly under hospital management were included.

Intervention: studies that evaluated interventions to implement guidelines were included. If the guidelines were aimed at multi-professional groups or other health care professionals, studies were only included if the results on medical health care professionals were reported separately, or medical health care professionals represented more than 50% of the target population. Studies that evaluated the introduction of guidelines targeted at undergraduate medical students were excluded.

Outcome: objective measures of provider behaviour, such as proportion of patients treated in accordance with guidelines. Only studies reporting dichotomous measures were included.

### Literature search

Studies were identified from a systematic review of guideline dissemination and implementation strategies across all settings[2]. Details of the search strategies and their development are described elsewhere[2]. Briefly, electronic searches were made of the following databases: Medline (1966–1998), HEALTHSTAR (1975–1998), Cochrane controlled trial register (4^th ^edition 1998), EMBASE (1980–1998), SIGLE (1980–1988) and the Cochrane Effective Practice and Organisation of Care group specialised register. For the review of interventions in all settings, over 150,000 hits were screened: 5000 were considered potentially relevant papers and full text articles of 863 were retrieved for assessment. In total, 235 studies were included in the systematic review of strategies across all settings. These studies were screened to identify potentially relevant studies for the hospital based review; we identified 108 studies conducted in hospital settings, of which 23 did not have a concurrent control group (were interrupted time series designs) and 32 other studies had continous measures. Therefore 53 of the 108 studies met our inclusion criteria.

### Data-extraction

The study followed the methods proposed by the Cochrane Effective Practice and Organisation of Care (EPOC) group[14]. Two independent reviewers extracted data on study design, methodological quality, participants, study settings, target behaviours, characteristics of interventions and study results, according to the EPOC checklist[15]. A second data extraction was done to assess potential organisational effect modifiers in hospital studies. Management support was regarded as positive if the manuscript gave information on direct support from the hospital management for the intervention, such as funding, or when the project was initiated by the hospital management or was set up as a result of hospital quality improvement strategies. "Academic hospital" was taken as the proxy for learning environment. Functional differentiation was operationalized by noting whether more than one specialty had been involved in the intervention, e.g. internal medicine and gynaecology, or whether more than one type of physician had been involved, e.g. specialists and residents, or when other professions, e.g. trained nurses, had been directly involved in the implementation process. Local consensus was regarded as being present when explicit information was given that the guidelines had been developed at the hospital or when major adaptations had been made to external guidelines before introduction at this hospital. Local consensus was also considered to be present when the implementation strategies had been developed at the hospital.

### Analysis

Analysis was based on the theoretical framework depicted in figure 1. The effect of the different intervention strategies on clinical outcomes was expected to be influenced by the organisational effect modifiers listed under the headings leadership, learning environment, functional differentiation and local consensus. Effects and modifiers may have different influences on clinical outcomes in inpatient or outpatient settings (figure 1).

In each comparison, the primary process of care measure was extracted, as defined by the authors. If multiple process of care measures were reported and none of them were defined as being the primary variable, effect sizes were ranked and the median value was taken. Effect sizes were constructed so that treatment benefits were denoted positively.

All statistical analyses were performed using the proc mixed procedure by SAS version 6.12. First we estimated the treatment effect (log odds ratio) and the variances in this effect for each comparison weighted for variance within the study and between studies. These estimated effects were used as responses in a random effect meta-regression model, in which we corrected for multiple comparisons in a single study. In most studies a unit of analysis error was found and insufficient data were presented to calculate cluster sizes. First we ignored the unit of analysis error to analyse all the studies included. Then, from the studies that reported sufficient data on the number of participants and professionals, a sensitivity analysis was performed. We did this by calculating the design effect by using the cluster sizes in each study and assuming a constant and conservative intracluster correlation of 0.20,[16] after which we re-ran the meta-regression model with and without a correction for a unit of analysis error.

To measure the effect of each individual intervention strategy, effect sizes were adjusted for other intervention components that appeared in at least one third of the studies on each strategy. Adjustment for other interventions that appeared less frequently was not possible due to small numbers. To evaluate the relative effectiveness of different intervention components, covariates were included in the model.

## Results

The 53 trials yielded 81 comparisons. The appendix gives an overview of these studies and the intervention components of each comparison compared to the intervention components, if present, in the control group [see Additional file 1]. The trials consisted of 39 randomised controlled trials (of which 32 were clustered randomised controlled trials), 7 controlled clinical trials and 7 controlled before-and-after studies; 19 were inpatients studies [17-36], 28 were outpatient studies [37-64] and 6 had mixed settings [65-70]. In the 81 comparisons, 22 involved a single intervention. Mean number of interventions per comparison was 2.5 (SD 1.3).

**Figure 2:**
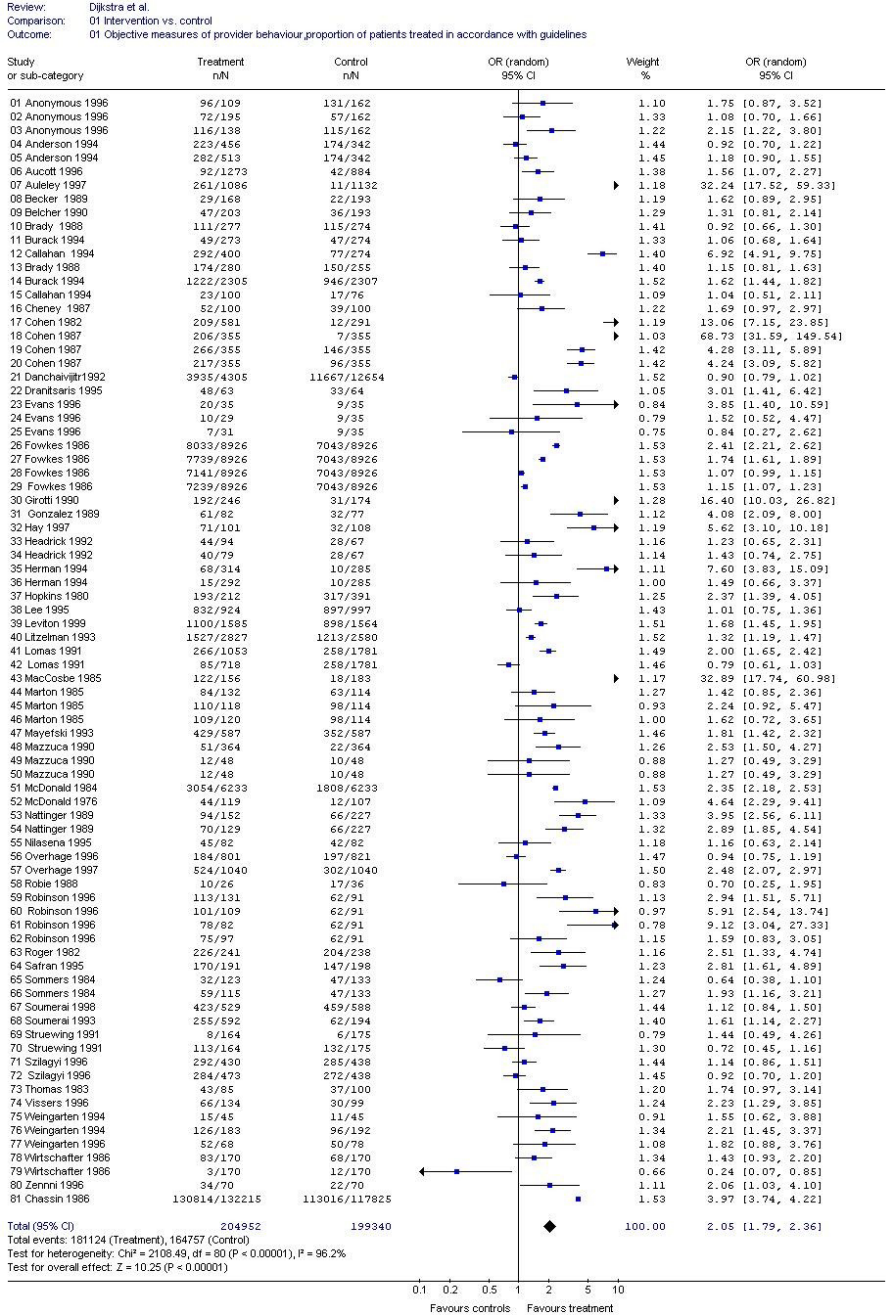
forest plot: total effect of guideline implementation in hospitals.

Table 1 shows the results of the meta-analysis on the effects of various intervention components. When taken together, the Odds ratio in all intervention strategies appeared to be 2.13 (SD 1.72–2.65). These total results are visualised in a Forest plot in figure 2. The Odds ratio for the forest plot is slightly different because we could not correct for different interventions within studies. Single interventions consisted of reminders or feedback; no other intervention strategies were applied as a single intervention strategy. Overall odds ratios were 2.18 in single intervention studies, versus 1.77 in studies that had more than one intervention component, the so-called multifaceted intervention studies. Intervention components applied most frequently were reminders, feedback, educational meetings and educational material. Only a few studies included outreach visits, consensus meetings, financial interventions or the role of an opinion leader. With regard to the specific components of individual intervention strategies, we found that all components showed positive effects, except for consensus meetings, outreach and financial interventions, possibly due to small numbers.

To learn more about the contribution of each intervention strategy to a multifaceted approach, we adjusted for co-operating intervention components to identify each unique contribution made by a specific intervention component when the other intervention components remained constant. Although adjustments could only be made for other intervention components that were co-operating in at least one third of the comparisons, we saw substantial changes in the results. The effects of educational material, reminders and feedback remained statistically significant, while the effects of educational meetings and patient-mediated interventions disappeared. The effect of the latter might be explained by the other co-operating intervention components. Furthermore, the revision of professional roles appeared to be a strong component in the intervention strategies besides organisational interventions, although the latter were not significant. Sensitivity analysis showed that, within the 47 comparisons in which cluster sizes could be calculated, adjustment for clustering effects showed some small changes in the effect sizes. There were no effect sizes that had become non significant due to adjustment for a design effect. Table 2 describes the effect of the different organisational factors on outcome measures. For most organisational effect modifiers, no significant differences were found in outcomes. Academic hospitals showed greater improvements in inpatient care only compared with community general hospitals.

However, in outpatient studies, community hospitals showed significantly larger effects. Furthermore, interventions that had not been developed internally, but had originated from outside the hospital, led to better outcomes, especially in outpatient studies.

## Discussion

This is the first systematic review to make an in depth exploration of guideline implementation in relation to the organisational characteristics of hospitals. Not only multifaceted interventions seemed to be effective, but also single interventions, contrary to our expectation that multifaceted interventions would prevail[69]. Single intervention strategies, particularly reminders, known to be effective in other settings, also appeared to be effective strategies in hospitals. Although a multi-faceted intervention including reminders may be effective, a single reminder strategy might provide a clearer or more consistent message and thus have more impact. Furthermore, educational material, reminders, feedback and revision of professional roles had more effect than other intervention strategies. We did not confirm our hypotheses on the influence of organisational factors, except for a learning environment in inpatient settings. Contrary to our expectations, effects were greater at community hospitals in outpatient studies. In some multi-centre studies, no difference was found between academic and community hospitals [27,28]. or only a moderate positive effect was found for academic hospitals[70]. External factors, such as the origin of the intervention, seemed to have more impact than internal factors.

This review of the research literature has the limitation that it was an explorative retrospective study that may have suffered from publication bias. There may also have been reporting bias, because different studies with different aims were brought together and compared, while their research question was not to measure the influence of contextual factors. Due to limited organisational data within the studies, the validity of the measurements of organisational effect modifiers can be challenged. Another limitation of the study concerned the validity of the determinants, e.g. with regard to teaching hospitals as a proxy for learning environment, when no other valid measurements for learning environment could be found in the manuscripts. Other potential effect modifiers had to be disregarded, because there were no data present, for example organisational slack[9]. Also the number of studies was limited, partly because studies were left out if they did not produce dichotomous measures or did not have a concurrent group (interrupted time series designs). Adding these studies might have given our analysis greater power and perhaps other conclusions. The analysis was not corrected for clustering effects, which may have led to an overestimation of the statistical significance of the effects. Finally the study is limited by the fact that the exhaustive search strategy and data extraction inhibited the inclusion of studies published after 1998. An update of this review in a new study is recommended, including the possibility of exploring the inclusion of interupted time series and studies with continous measures.

Theories on organisational effect modifiers are mostly based on study results from a wide range of fields inside and outside health care and when they are based on health care, this mostly concerns primary care. Despite the pertinence of regarding organisational factors as being crucial for quality improvement, there is only limited research evidence for the claims made. The influence of possible organisational effect modifiers on the quality of care at hospitals needs more research attention and more evidence is needed from inside not outside hospitals for the development of theories in this field. Ongoing research should develop validated measures of potentially important organisational constructs and explore their influence on quality of care.

## Conclusion

There is no 'magic bullet' in terms of the most effective strategy or organisational effect modifiers for the implementation of change within hospitals. On an organisational level, barriers against and facilitators for effective interventions are unclear. Depending on the management policy and other local factors, such as funds available and motivation of the health care personnel, hospitals might wish to focus on building a learning organisation [71] or on adopting proven, effective, strategies from outside.

## Competing interests

The author(s) declare that they have no competing interests.

## Authors' contributions

RD, MW, JB and RG drew up the design and framework of this manuscript. Data extraction on profesional performances and intervention strategies was done by RT, JG, RD and MW. Data extraction on organisational features was done by RD and MW. Statistical analysis was done bij RA. All authors read and approved the final manuscript.

## Pre-publication history

The pre-publication history for this paper can be accessed here:



## Supplementary Material

Additional File 1 Studies to implement guidelines at hospitals. The reviewed studies are listed with details on type of study, setting, target, number of patientes, intervention strategies and post study percentages on the primary outcome measure. Click here for file

## Figures and Tables

**Table 1:** Effectiveness of specific intervention components

	**n**	**Total comparisons OR (95%CI)**	
Total effect	81	2.13 (1.72–2.65)	
Single interventions	22	2.18 (1.62–2.94)	
Multifaceted interventions	59	1.77 (1.36–2.31)	
			
**Single intervention strategies**			
Reminders	18	2.14 (1.49–3.07)	
Feedback	4	2.33 (1.32–4.10)	

**Components of multifaceted intervention strategies**	**n**	**Unadjusted Odds ratios OR (95%CI)**	**Adjusted Odds ratios^# ^OR (95%CI)**

Educational meeting (ab)	34	1.98 (1.39–2.82)	0.66 (0.25–1.76)
Educational material (bc)	41	2.13 (1.60–2.84)	1.84 (1.09–3.11)
Consensus meeting (ad)	6	1.35 (0.73–2.49)	0.76 (0.56–1.04)
Reminders (ac)	54	2.10 (1.61–2.75)	1.92 (1.39–2.65)
Feedback (ac)	20	2.01 (1.28–3.18)	2.50 (1.38–4.52)
Patient mediated (abc)	12	2.00 (1.08–3.70)	0.64 (0.16–2.54)
Outreach	2	1.67 (0.70–4.01)	Na
Opinion leader	5	1.51 (1.13–2.02)	na
Revision of professional roles (abc)	13	2.57 (1.13–5.87)	9.78 (3.22–29.70)
Financial	3	3.16 (0.40–24.9)	Na
Organisational (abce)	15	1.89 (1.12–3.20)	8.41 (0.81–87.2)

n: number of comparisons, OR: Odds ratios (95% confidence intervals) of intervention versus control

^# ^adjusted for other intervention components if present in > 1/3 of the comparisons.

Adjusted for (a): educational material, (b): reminders (c): educational meeting (d): feedback (e): revision of professional roles, na: not available due to small numbers.

**Table 2:** Effects of the presence of organisational features to improve the quality of care at hospitals

	**Total comparisons**		**Inpatient**		**Outpatient**	
	
**Organisational effect modifiers**	**n**	**OR (95%CI)**	**n**	**OR (95%CI)**	**n**	**OR (95%CI)**
**leadership**						
management support	20	1.95 (1.26–3.03)	5	2.77 (0.90–8.51)	11	1.47 (0.83–2.62)
no management support	61	2.20 (1.71–2.84)	24	1.98 (1.21–3.24)	30	2.66 (1.94–3.65)
**learning environment**						
Academic hospital	44	2.15 (1.62–2.86)	9	3.42 (2.25–5.19)	32	1.91 (1.41–2.59)
non academic hospital	37	2.11 (1.49–2.98)	20	1.44 (0.95–2.19)*	9	3.90 (2.87–5.29)*
**functional differentiation**						
>1 medical speciality involved	23	1.57 (1.03–2.38)	13	1.79 (0.90–3.54)	5	1.33 (0.41–4.36)
1 medical speciality involved	58	2.39 (1.87–3.06)	16	2.48 (1.39–4.41)	36	2.43 (1.80–3.27)
						
more physician types	38	2.06 (1.53–2.79)	15	1.97 (1.15–3.37)	21	2.35 (1.52–3.61)
one physician type	43	2.22 (1.61–3.06)	14	2.62 (1.20–5.76)	20	2.21 (1.46–3.34)
						
multiprofessional task	14	1.64 (0.94–2.88)	3	1.46 (0.09–24.0)	9	1.86 (0.93–3.71)
no multiprofessional task	67	2.25 (1.78–2.85)	26	2.25 (1.44–3.53)	32	2.41 (1.74–3.34)
**local consensus**						
own guideline	45	1.99 (1.49–2.65)	19	2.11 (1.24–3.60)	23	2.08 (1.41–3.06)
external guideline	36	2.35 (1.68–3.30)	10	2.26 (0.99–5.18)	18	2.57 (1.63–4.06)
						
own intervention	59	1.78 (1.36–2.32)	16	2.56 (1.56–4.21)	36	1.85 (1.32–2.60)
external intervention	22	4.62 (2.82–7.57)**	13	1.37 (0.60–3.11)	5	12.7 (4.99–32.2)**

n: number of comparisons; OR: Odds ratios (95% confidence interval)

significant differences between presence or absence of possible moderators: * p < 0.05 ** p < 0.01
