# Supplementary material for: The relationship between organisational characteristics and the effects of clinical guidelines on medical performance in hospitals, a meta-analysis
Source: BMC Health Serv Res. 2006 Apr 28;6:53. doi: 10.1186/1472-6963-6-53 (PMC1479332; doi:10.1186/1472-6963-6-53)
Supplement: Additional File 1 — Studies to implement guidelines at hospitals. The reviewed studies are listed with details on type of study, setting, target, number of patientes, intervention strategies and post study percentages on the primary outcome measure. [file 1472-6963-6-53-S1.doc]

Appendix 1 Studies to implement guidelines at hospitals

| **Author** | **Year** | **Type** | **Setting** | **Target** | **Compa-rison** | **Number of patients** | **Intervention strategy** | | **Post intervention** | |
| --- | --- | --- | --- | --- | --- | --- | --- | --- | --- | --- |
|  |  |  |  |  |  |  | **treatment**  **group** | **control group** | **% treatment** | **% control** |
| Anonymous[66] | 1996 | cba | mixed | Treatment of benign prostatic hyplasia | 1 | 271 | ma re co mm | ma re | 88 | 81 |
|  |  |  |  | History taken according to score form | 2 | 357 | ma re ol mm or | - | 37 | 35 |
|  |  |  |  | Urinalysis | 3 | 300 | ma re co mm or | - | 84 | 71 |
| Anderson[29] | 1994 | rct | in | Prophylaxis for trombolism | 1 | 798 | ma me re fb or | - | 49 | 51 |
|  |  |  |  |  | 2 | 855 | ma me re or | - | 55 | 51 |
| Aucott [38] | 1996 | rct | out | Hypertension treatment | 1 | 2157 | ma me fb ol or | - | 7,20 | 4,70 |
| Auleley[64] | 1997 | rct | mixed | Reduction of radiography ankle injuries | 1 | 2218 | ma me re | - | 76 | 99 |
| Becker [55] | 1989 | rct | out | Measurement of occular pressure | 1 | 361 | re pm mm | - | 17,20 | 11,20 |
|  |  |  |  | Cervical smear done | 2 | 396 | re mm | - | 23,10 | 18,40 |
| Belcher [58] | 1990 | rct | out | Influenza vaccinations | 1 | 551 | me re | - | 40 | 42 |
|  |  |  |  | Fecal occult blood testing | 2 | 547 | re pm | - | 18 | 17 |
|  |  |  |  | Recoding smoking status | 3 | 674 | mm or | - | 73 | 28 |
| Brady [61] | 1988 | rct | out | Influenza vaccinations | 1 | 535 | ma me fb | - | 62 | 59 |
| Burack [69] | 1994 | rct | mixed | Mammography | 1 | 4612 | me re mm fi | me mm fi | 53 | 41 |
| Callahan [52] | 1994 | rct | out | Treatment of depression | 1 | 175 | ma re pm or | me | 23 | 22 |
| Chassin[67] | 1986 | rct | mixed | Reduction of x ray pelvimetry | 1 | 249500 | ma me fb | - | 1,06 | 3,64 |
| Cheney [39] | 1987 | rct | out | 10 preventive health measures | 1 | 200 | re | - | 52 | 39 |
| Cohen [40] | 1982 | rct | out | Influenza vaccinations | 1 | 872 | me re ma me re fi | me | 36 | 4 |
| Cohen [41] | 1987 | rct | out | Stop smoking treatment | 1 | 710 | ma me re pm | ma me | 58 | 2 |
|  |  |  |  |  | 2 | 710 | ma me re | ma me | 75 | 41 |
|  |  |  |  |  | 3 | 710 | ma me re fi pm | ma me | 61 | 27 |
| Danchaivijitr  [34] | 1992 | rct | in | Urethral catheterisation | 1 | 16959 | re | - | 8,60 | 7,80 |
| Dranitsaris[33] | 1995 | rct | in | Oncology, treatment | 1 | 127 | ma fb | ma | 76 | 51,60 |
| Evans [70] | 1996 | rct | mixed | Treatment of hypercholesterolaemie | 1 | 70 | ma me re pm | me | 58 | 25 |
|  |  |  |  |  | 2 | 64 | re pm | me | 36 | 25 |
|  |  |  |  |  | 3 | 66 | ma me | me | 24 | 25 |
| Fowkes [24] | 1986 | Cba | in | Reduction of preoperative chest X ray | 1 | 17852 | ma re or | - | 10 | 21,10 |
|  |  |  |  |  | 2 | 17852 | ma fb | - | 13,30 | 21,10 |
|  |  |  |  |  | 3 | 17852 | ma re | - | 20 | 21,10 |
|  |  |  |  |  | 4 | 17852 | ma rp or | - | 18,90 | 21,10 |
| Girotti [35] | 1990 | cba | in | Antibiotic prescribing | 1 | 420 | re | ma | 78 | 18 |
| Gonzalez [60] | 1989 | rct | out | Preventive stool tests | 1 | 159 | re | - | 74 | 41 |
| Hay[17] | 1997 | cct | in | Guidelines on length of stay | 1 | 209 | re | - | 70 | 30 |
| Headrick [57] | 1992 | rct | out | Compliance to cholesterol guidelines | 1 | 161 | me re pm | me | 46,80 | 41,80 |
|  |  |  |  |  | 2 | 146 | me re | me | 50,60 | 41,80 |
| Herman [45] | 1994 | rct | out | Pneumococcal vaccination | 1 | 599 | ma me pm rp | ma me | 21,60 | 3,40 |
|  |  |  |  | Influenza vaccination | 2 | 577 | ma me pm | ma me | 5,10 | 3,40 |
| Hopkins [62] | 1980 | cct | mixed | Reduction of secondary operation | 1 | 603 | ma me | - | 9 | 19 |
| Lee [65] | 1995 | cct | mixed | Reducion of admission to coronary care unit | 1 | 1921 | re | - | 10 | 10 |
| Leviton[19] | 1999 | rct | in | Prescribing antenatal corticosteroids | 1 | 3149 | ma me re fb or ol co | ma | 69,40 | 57,40 |
| Litzelman[42] | 1993 | rct | out | Diabetes referral to podiatrist | 1 | 5407 | re | re | 54 | 47 |
| Lomas[32] | 1991 | rct | in | Vaginal birth after previous cesarean section | 1 | 2834 | ma me ol | ma | 25,30 | 14,50 |
|  |  |  |  |  | 2 | 2499 | fb co | ma | 11,80 | 14,50 |
| MacCosbe[36] | 1985 | rct | in | Antibiotic treatment | 1 | 339 | fb rp | - | 78 | 10 |
| Marton[53] | 1985 | rct | out | Reduction of glucose test ordering | 1 | 246 | ma me fb | - | 36 | 45 |
|  |  |  |  | Reduction of digoxin test ordering | 2 | 232 | fb | - | 7 | 14 |
|  |  |  |  | Reduction of digoxin test ordering | 3 | 234 | ma me | - | 9 | 14 |
| Mayefski[46] | 1993 | rct | out | Child care, recording history | 1 | 1174 | fb | - | 73 | 60 |
| Mazzuca[54] | 1990 | cba | out | Diabetes self monitoring guidelines | 1 | 728 | ma me re or | ma me | 14 | 6 |
|  |  |  |  | Diabetes guidelines on oral drugs | 2 | 96 | ma me re or | ma me | 26 | 20 |
|  |  |  |  |  | 3 | 96 | ma me re | ma me | 24 | 20 |
| McDonald[43] | 1984 | rct | out | Guidelines on preventive care | 1 | 12466 | re | - | 49 | 29 |
| McDonald [51] | 1976 | rct | out | Guidelines on test ordering | 1 | 226 | re | - | 36,90 | 11,20 |
| Nattinger [48] | 1989 | cba | out | Mammography | 1 | 379 | fb | - | 61,80 | 29,20 |
|  |  |  |  |  | 2 | 356 | re pm | - | 54,30 | 29,20 |
| Nilasena [49] | 1995 | rct | out | Diabetes guidelines | 1 | 164 | me re | me | 54,90 | 51 |
| Overhage [31] | 1996 | rct | in | Preventive care actions | 1 | 1622 | re | - | 23 | 24 |
| Overhage [18] | 1997 | rct | in | Following corollary orders | 1 | 2080 | ma re | ma | 50,40 | 29 |
| Robie [47] | 1988 | cba | out | Preventive stool tests | 1 | 62 | me re | - | 40 | 46 |
| Robinson [25] | 1996 | cba | in | Trombolytic therapy | 1 | 222 | ma fb co | - | 86 | 68 |
|  |  |  |  |  | 2 | 200 | ma re fb | - | 93 | 68 |
|  |  |  |  |  | 3 | 173 | ma re fb | - | 95 | 68 |
|  |  |  |  |  | 4 | 188 | ma fb | - | 77 | 68 |
| Rogers [56] | 1982 | rct | out | Hypertension, omitting potassium test | 1 | 479 | re | - | 6,10 | 14,10 |
| Safran [59] | 1995 | rct | out | Guideline on blood testing | 1 | 389 | re | - | 89 | 74 |
| Sommers [30] | 1984 | rtc | in | Management of anemia | 1 | 256 | fb co | - | 26 | 35 |
|  |  |  |  |  | 2 | 248 | fb | - | 51 | 35 |
| Soumerai [26] | 1998 | rct | in | Treatment of myocardial infarction | 1 | 1117 | ma me ol rp | ma | 80 | 78 |
| Soumerai [28] | 1993 | rct | in | Guidelines on blood transfusion | 1 | 786 | ma me ou | - | 43 | 32 |
| Struewing [37] | 1991 | cct | out | Cancer screening by sigmoidoscopy | 1 | 339 | me re rp or | - | 4,70 | 3,20 |
|  |  |  |  | Cancer screening by rectal examination | 2 | 339 | me re rp or | - | 68,80 | 75,40 |
| Szilagyi [68] | 1996 | rct | mixed | Guidelines on immunizations | 1 | 868 | re | - | 68 | 65 |
|  |  |  |  |  | 2 | 911 | re pm | pm | 60 | 62 |
| Thomas [44] | 1983 | rct | out | Guidelines on ambulatory care | 1 | 185 | re | - | 50,25 | 37,30 |
| Vissers [63] | 1996 | rct | mixed | Guidelines on treatment behaviour | 1 | 233 | ma re | ma | 49 | 30 |
| Weingarten[20] | 1994 | cct | in | Guidelines on congestive heart failure | 1 | 90 | re | - | 33 | 25 |
| Weingarten [21] | 1994 | cct | in | Guidelines on chest pain | 1 | 375 | re | - | 69 | 50 |
| Weingarten [23] | 1996 | cct | in | Guidelines on treatment of pneumonia | 1 | 146 | re | - | 76 | 64 |
| Wirtschafter[27] | 1986 | rct | in | Neonatal chest X rays | 1 | 340 | ma me | ma | 49 | 40 |
|  |  |  |  | Monitoring of infant blood pressure | 2 | 340 | ma me | ma | 2 | 7 |
| Zenni [50] | 1996 | rct | out | Guidelines on childhood development | 1 | 140 | re | - | 48 | 31 |

cba: controlled before and after study, rct: randomised controlled trial, cct: controlled clinical trial,

Intervention or control strategies: ma: educational material, me: educational meeting, re: reminders, fb: feedback, ou: outreach visits, pm: patient mediated, ol: opinion leader, co: consensus meeting, rp: revision of professional roles, fi: financial, or: organisational.
